# Delayed brain ischemia tolerance induced by electroacupuncture pretreatment is mediated via MCP-induced protein 1

**DOI:** 10.1186/1742-2094-10-63

**Published:** 2013-05-10

**Authors:** Zhuqing Jin, Jian Liang, Jing Wang, Pappachan E Kolattukudy

**Affiliations:** 1 School of Basic Medicine, Zhejiang Chinese Medical University, Hangzhou 310053, Zhejiang, China; 2 Burnett School of Biomedical Sciences, University of Central Florida College of Medicine, 4000 Central Florida Boulevard, Orlando, FL 32816, USA

**Keywords:** Ischemic stroke, Electroacupuncture, Monocyte chemotactic protein-induced protein 1 (MCPIP1), Middle cerebral artery occlusion (MCAO), Proinflammatory cytokines

## Abstract

**Background:**

Emerging studies have demonstrated that pretreatment with electroacupuncture (EA) induces significant tolerance to focal cerebral ischemia. The present study seeks to determine the involvement of monocyte chemotactic protein-induced protein 1 (MCPIP1), a recently identified novel modulator of inflammatory reactions, in the cerebral neuroprotection conferred by EA pretreatment in the animal model of focal cerebral ischemia and to elucidate the mechanisms of EA pretreatment-induced ischemic brain tolerance.

**Methods:**

Twenty-four hours after the end of the last EA pretreatment, focal cerebral ischemia was induced by middle cerebral artery occlusion (MCAO) for 90 minutes in male C57BL/6 mice and MCPIP1 knockout mice. Transcription and expression of MCPIP1 gene was monitored by qRT-PCR, Western blot and immunohistochemistry. The neurobehavioral scores, infarction volumes, proinflammatory cytokines and leukocyte infiltration in brain and NF-κB signaling were evaluated after ischemia/reperfusion.

**Results:**

MCPIP1 protein and mRNA levels significantly increased specifically in mouse brain undergoing EA pretreatment. EA pretreatment significantly attenuated the infarct volume, neurological deficits, upregulation of proinflammatory cytokines and leukocyte infiltration in the brain of wild-type mice after MCAO compared with that of the non-EA group. MCPIP1-deficient mice failed to evoke EA pretreatment-induced tolerance compared with that of the control MCPIP1 knockout group without EA treatment. Furthermore, the activation of NF-κB signaling was significantly reduced in EA-pretreated wild-type mice after MCAO compared to that of the non-EA control group and MCPIP1-deficient mice failed to confer the EA pretreatment-induced inhibition of NF-κB signaling after MCAO.

**Conclusions:**

Our data demonstrated that MCPIP1 deficiency caused significant lack of EA pretreatment-induced cerebral protective effects after MCAO compared with the control group and that MCPIP1 is involved in EA pretreatment-induced delayed brain ischemia tolerance.

## Background

Stroke is the second leading cause of death and the most frequent cause of permanent disability worldwide [1]. In recent investigation, it is reported that stroke strikes approximately 800,000 people per year in the United States alone, killing about 150,000 [2,3]. Ischemic strokes are more prevalent than hemorrhagic. Thrombosis, an embolism or systemic hypoperfusion can cause an ischemic stroke. Within the core of the ischemic brain, where blood flow is most severely restricted, excitotoxic and necrotic cell death occurs within minutes. In the periphery of the ischemic area, where collateral blood flow can buffer the full effects of the stroke, ischemic brain damage happens within hours to days post stroke via mechanisms such as apoptosis and inflammation [4,5]. Inflammatory mechanisms that are activated within hours after brain ischemia represent a key target of current translational ischemic stroke research [6]. It has been reported that the levels of proinflammatory cytokines and chemokines increased after focal ischemia. Chemokines are cytokines that have the ability to induce chemotaxis on neighboring cells, particularly those involved in inflammatory actions [7]. While some cytokines may offer protection, many cytokines and most chemokines have been shown to participate in the neuronal damage processes [8,9].

A brief exposure to sublethal or noninjurious stimuli renders the brain resistant to a subsequent damaging ischemic insult. Ischemic tolerance consists of an early phase that occurs within minutes after induction, followed by a delayed phase that develops many hours or even days later. Delayed preconditioning has generated much interest because it produces a potent neuroprotection whose mechanisms may suggest new treatments for ischemic stroke [10]. Published data have shown recently that electroacupuncture (EA) pretreatment has such a neuroprotective effect [11-17]. EA pretreatment-induced neuroprotection or bioprotection is related to the suppression of the inflammatory response in the ischemic or injured area, but the mechanisms involved in the protection afforded by EA pretreatment are poorly understood [18-21].

Monocyte chemotactic protein-induced protein 1 (MCPIP1, also known as ZC3H12A) is a recently identified protein in human peripheral blood monocytes treated with monocyte chemotactic protein-1 (MCP-1) [22]. In our previous studies, MCPIP1 was shown to be a negative regulator of macrophage activation [23]. Further investigations by our group and others indicated that MCPIP1 can play a significant anti-inflammatory role by inhibiting the generation of a set of major proinflammatory cytokines [24,25]. In our previous studies, MCPIP1 was also found to be inducibly expressed in monocytes, macrophages, and endothelial cells with lipopolysaccharide (LPS) stimulation [26,27] and participates in LPS preconditioning-induced ischemic brain tolerance [28]. The present study examined the effects of EA pretreatment at the Baihui acupoint on MCAO-induced cerebral inflammatory response and ischemic brain injury in wild-type and MCPIP1 knockout mice.

## Methods

### Animals

MCPIP1 knockout mice were established as previously described [24]. Briefly, Mcpip1^−/−^ mice were generated by homologous recombination in embryonic stem cells from C57/BL6 background mice. Exons 3, 4, 5 and most part of 6 of mouse Mcpip1 were targeted with a LacZ-neomycin cassette in embryonic stem cells established from C57/BL6 mice and established Mcpip1^−/−^ mice in pure C57/BL6 background. The absence of MCPIP1 protein in Mcpip^−/−^ mice was confirmed by immunoblotting. Six to eight-week-old mice were used. All experimental procedures were approved by the Institutional Animal Care and Use Committee of the University of Central Florida. We performed all the experiments by using littermate mice.

### Electroacupuncture pretreatment

**Figure 1:**
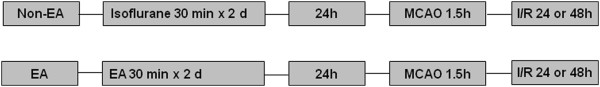
**Diagram of experimental protocol.** Non-EA, animals received no EA treatment but isoflurane inhalation the same as the EA group; EA, preconditioning with EA for 30 min daily for two consecutive days before MCAO. EA, electroacupuncture; MCAO, middle cerebral artery occlusion.

The experimental protocol is shown in Figure 1. EA pretreatment was performed following a modification of the method reported by Wang and colleagues [15,29] at the acupoint ‘Baihui (GV 20)’, which is located at the intersection of the sagittal midline and the line linking the two ears. Briefly, animals were anesthetized with isoflurane (induction with 3%; maintenance with 1.2%) in oxygen-enriched air by face mask, and rectal temperature was controlled at 37 ± 0.5°C throughout the experiment with heating lamps. The acupoint ‘Baihui (GV 20)’ was stimulated at an intensity of 1 mA and a frequency of 2/15 Hz for 30 min, using the Hwato Electronic Acupuncture Treatment Instrument (Model No. SDZ-V, Suzhou Medical Appliances Co., Ltd., Suzhou, China). The animals received daily EA preconditioning for two consecutive days, and were subjected to MCAO 24 h after the end of the last EA pretreatment. The mice in the control group underwent the same procedures as the EA group without the electroacupuncture. All electroacupuncture experimental procedures were approved by the Institutional Animal Care and Use Committee of the University of Central Florida. Protocol number is 10-31 (2012).

### Mice focal brain ischemia reperfusion model

For focal brain ischemia, mouse transient middle cerebral artery occlusion (MCAO) was produced by filament occlusion of the right MCA as previously described [28]. In brief, mice were anesthetized with isoflurane (induction with 3%; maintenance with 1.2%) in oxygen-enriched air by face mask, and rectal temperature was controlled at 37 ± 0.5°C throughout the experiment with heating lamps. Unilateral MCAO was performed by inserting a 7–0 nylon monofilament into the internal carotid artery via an external carotid artery stump and then positioning the filament tip for occlusion at a distance of 8 to 9 mm beyond the internal carotid/pterygopalatine artery bifurcation. MCA was occluded for 90 min followed by reperfusion.

### Brain infarction measurement

The brains were stained with 2,3,5-triphenyltetrazolium chloride (TTC) (Sigma-Aldrich, St Louis, MO, USA) to determine infarct volume [28]. After 90 min of MCAO and 48 h of reperfusion, mice were anesthetized with 4% isoflurane and brains were removed and sectioned coronally at a thickness of 2 mm and incubated in 2% TTC at 37°C for 20 min. Brain slices were then fixed in 4% paraformaldehyde at 4°C overnight and scanned into a computer, and quantified using the Image J software (NIH, Bethesda, MD, USA). Infarct volume was expressed as a percentage of the contralateral hemisphere. There were 10 mice in each group.

### Neurological function assessment

The functional outcome of the animals were assessed at 48 h after ischemic/reperfusion. A modified Bederson score [30,31] was determined according to the following scoring system: 0, no deficit; 1, forelimb flexion; 2, as for 1, plus decreased resistance to lateral push; 3, unidirectional circling; 4, longitudinal spinning or seizure activity; 5, no movement. Twenty-four hours and 48 h after surgery, the foot fault test and grip test were performed. The grip test, also known as the string test, was adapted from a published report [32], with a modified scoring system. For this test, the mouse was placed midway on a string between two supports and rated as follows: 0, falls off; 1, hangs onto string by one or both forepaws; 2, as for 1, and attempts to climb onto string; 3, hangs onto string by one or both forepaws plus one or both hind paws; 4, hangs onto string by fore- and hind paws plus tail wrapped around string; 5, escape (to the supports).

### Quantitative real-time PCR

Quantitative real-time polymerase chain reaction (qRT-PCR) was performed as previously described [29]. Briefly, total RNA was isolated using RNA STAT-60 reagent (Tel-Test, Inc., Friendswood, TX, USA), after removing the genomic DNA using DNase I (Ambion, Austin, TX, USA), 2.0 ug of total RNA from microglia or mouse brain tissue was reverse-transcribed to cDNA using a commercially available kit (Applied Biosystems, Foster City, CA, USA). qRT-PCR was performed with an iCycler Thermal Cycler (Bio-Rad, Hercules, CA, USA) using 2 × SYBR Green master mixes (Bio-Rad). Forty cycles were conducted as follows: 95°C for 30s, 60°C for 30s, proceeded by 10 min at 95°C for polymerase activation. Quantification was performed by the delta cycle time method, with mouse β-actin used for normalization. Human MCPIP1 gene-specific primers (Integrated DNA Technologies, Coralville, IA, USA) were F: 5´-GCCGGCGGCCTTA; R: 5´-GCACTGCTCACTCTCTGTTAGCA. The mouse-specific primers (Integrated DNA Technologies) are as follows, MCPIP1: F: 5’-CCCCCTGACGACCCTTTAG; R: 5’ GGCAGTGGTTTCTTACGAAGGA, tumor necrosis factor-alpha (TNF-α): F: 5’- CTGAGGTCAATCTGCCCAAGTAC; R: 5’-CTTCACAGAGCAATGACTCCAAAG, interleukin-1 beta (IL-1β): F: 5’- GCCCATCCTCTGTGACTCAT; R: 5’- AGGCCACAGGTATTTTGTCG, IL-6: F: 5’- TCGTGGAAATGAGAAAAGAGTTG; R: 5’- AGTGCATCATCGTTGTTCATACA, MCP-1: F: 5’- CCATCTCTGACCTGCTCTTCCT; R: - AGACCCACTCATTTGCAGCAT, beta-actin: F: 5’- AAATCGTGCGTGACATCAAAGA, R: 5’- GGCCATCTCCTGCTCGAA.

### Western blot

Western blot was performed as previously described [28]. Proteins from mouse brain tissue were extracted and concentrations were determined by the Bradford method (Bio-Rad) with bovine serum albumin as the standard. Proteins (50 ug) were separated by SDS-PAGE and transferred onto nitrocellulose membranes in transfer buffer containing 0.1% SDS. The membranes were blocked with 5% nonfat dry milk in 0.05% Tween 20 in Tris-buffered saline (TTBS) for 2 h and incubated with the primary antibodies against MCPIP1 (Santa Cruz Biotechnology, Santa Cruz, CA, USA), phosphor-p65 (Cell Signaling Technology, Danvers, MA, USA), p65 (Cell Signaling Technology) at a 1:1000 dilution in the blocking buffer, 4°C, gently shaking, overnight. After being washed with TTBS three times for 10 min each, the membranes were incubated with a 1:2,000 dilution of secondary antibody (Santa Cruz Biotechnology) in TTBS for 1 h. Following three 10-min washes with TTBS, membranes were incubated with SuperSignal West Pico Chemiluminescent Substrate (Thermo Scientific Pierce Antibodies, Rockford, IL, USA) and exposed to X-ray film. The intensity of bands was quantified by AlphaImager 2200 (Alpha Innotech, San Leandro, CA, USA). The ratios between interested protein bands and the loading control (β-actin, total p65) were calculated and the data are expressed as the normalized folds with respect to sham.

### Immunohistochemistry

At 48 h after reperfusion, mice were transcardially perfused under anesthesia with ice-cold phosphate-buffered saline and then with 4% paraformaldehyde. Brains were removed and fixed overnight in 4% paraformaldehyde at 4°C. The brains were sectioned coronally at 30 μm thickness in ice-cold phosphate-buffered saline using a vibrating microtome (Leica Microsystems, Buffalo Grove, IL, USA). The sections were placed in an anti-freeze solution and stored at −20°C for later use. They were washed, the nonspecific binding sites on sections were blocked with 3% bovine serum albumin, and incubated overnight with primary antibody against CD45, leukocyte common antigen, antibody against glial fibrillary acidic protein (GFAP), primary antibody against CD11b (BD Biosciences Pharmingen, San Diego, CA, USA), primary antibody against neuron-specific enolase (NSE) (Life Technologies, Carlsbad, CA, USA), primary antibody against MCPIP1 (Santa Cruz Biotechnology, CA, USA), AlexaFluor™ 488-conjugated secondary antibody (Life Technologies) and AlexaFluor™ 594-conjugated secondary antibody (Life Technologies) and scanned under a fluorescence microscope (Leica TCS SP5) at 400× magnification. Three fields per section were captured and analyzed.

### Statistical analysis

The data are presented as mean ± standard deviation (SD). Multiple comparisons were evaluated by one-way ANOVA followed by the Tukey or Dunnett test. Two-group comparisons were analyzed by the two-tailed Student *t* test. For all analyses, a value of *P* <0.05 was considered significant.

## Results

### MCPIP1 induction in mouse brain by EA pretreatment

**Figure 2:**
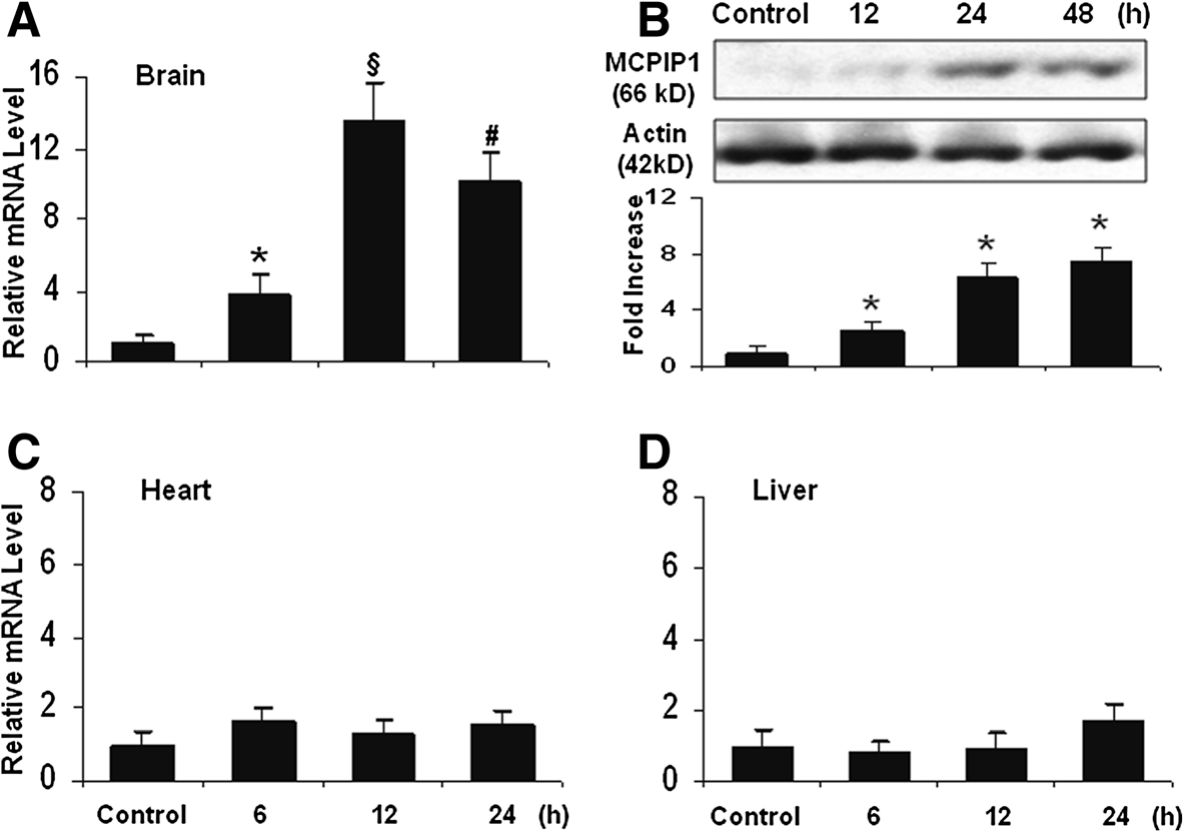
**EA treatment induced MCPIP1 in the brain. (A)** MCPIP1 mRNA expression in mouse brain by EA treatment as measured by qRT-PCR. Values represent mean ± SD, *P <0.05, #*P* <0.01, §*P* <0.001 versus control. **(B)** MCPIP1 protein levels in mouse brain by EA treatment as measured by Western blot. Results are representative of three independent experiments. *P <0.05 versus control. **(C)** MCPIP1 mRNA expression in mouse heart by EA treatment as measured by qRT-PCR. Values represent mean ± SD. **(D)** MCPIP1 mRNA expression in mouse liver by EA treatment as measured by qRT-PCR. Values represent mean ± SD. EA, electroacupuncture; MCPIP1, monocyte chemotactic protein-induced protein 1; qRT-PCR, quantitative real-time polymerase chain reaction; SD, standard deviation.

In ischemic stroke, EA pretreatment has been demonstrated to have a significant protective effect against brain damage, but the underlying mechanism remains poorly understood. First, we examined whether EA pretreatment induces MCPIP1 in mouse brain. The MCPIP1 mRNA level in mouse cortex brain was significantly induced by EA pretreatment compared to controls; significant increase of MCPIP1 in transcript level was detected at 6 h and reached 13.6- ± 2.13 fold at 12 h after EA pretreatment (*P* <0.001; Figure 2A). Consistently, the MCPIP1 protein levels in mouse brain were significantly higher in EA pretreatment than in the controls, 7.5- ± 0.95 fold at 48 h after EA pretreatment (*P* <0.05; Figure 2B). To determine whether EA treatment induces the expression of MCPIP1 systemically after EA treatment, we measured the transcript level of MCPIP1 in heart and liver tissue. The results showed that there was no significant induction of MCPIP1 in the heart (Figure 2C) and liver (Figure 2D) after EA treatment compared with the control group. Thus, the EA treatment we used induced MCPIP1 expression selectively in the brain.

**Figure 3:**
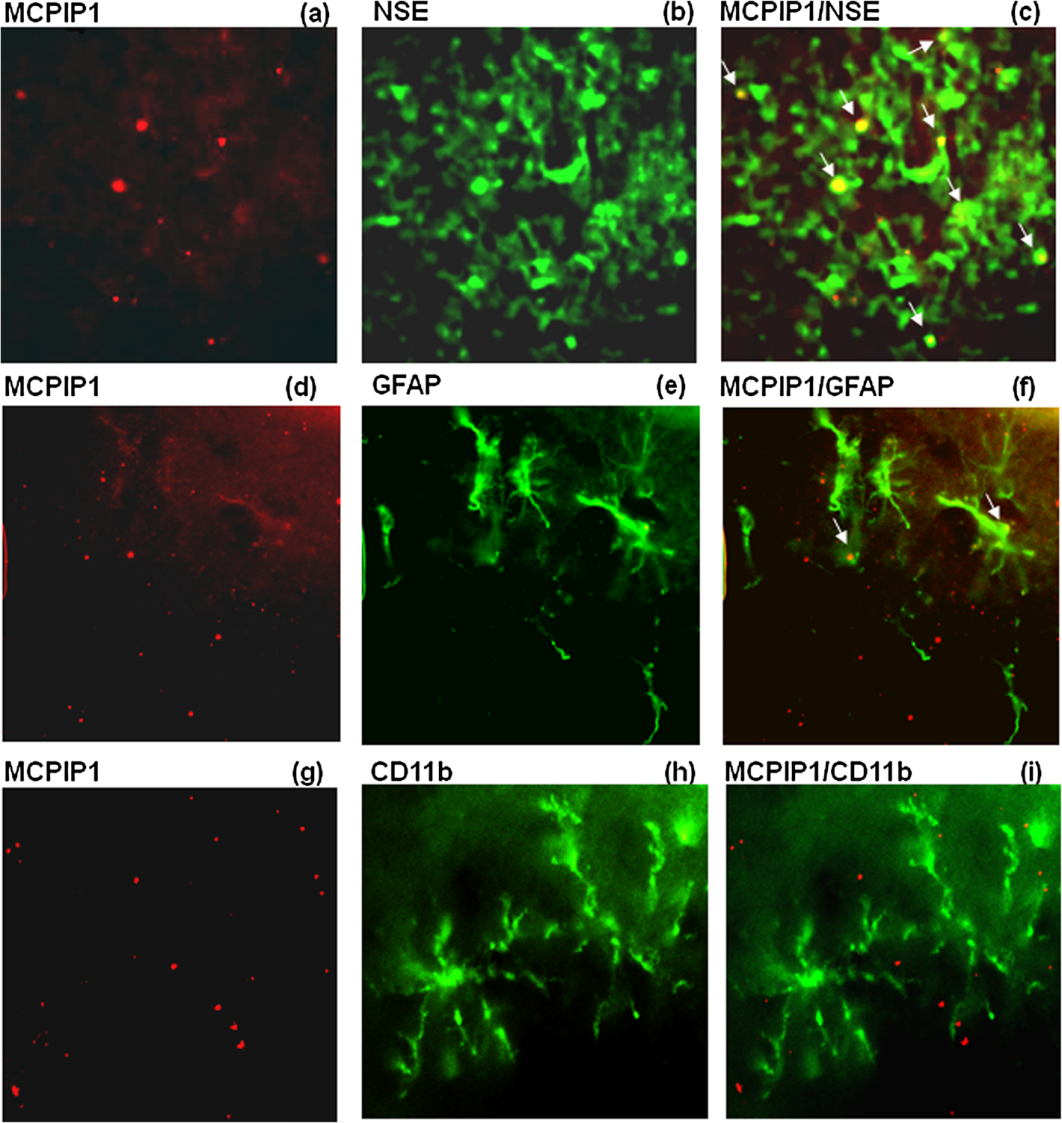
**MCPIP1 is expressed in neurons and astrocytes.** Co-localization of MCPIP1 expression of **(a)** NSE, **(d)** GFAP, and **(g)** CD11b at 24 h after the end of the last EA pretreatment. Yellow fluorescence indicates co-localization of MCPIP1/NSE **(c)** and MCPIP1/GFAP **(i)**. MCPIP1/CD11b **(f)** showed very few yellow fluorescent structures. Similar results were obtained from a total of 12 mice (n = 4 per group). (Magnification 400X). EA, electroacupuncture; GFAP, glial fibrillary acidic protein; MCPIP1, monocyte chemotactic protein-induced protein 1; NSE, neuron-specific enolase.

To determine the cellular localization of MCPIP1 expression, sections from mice brain at 24 h after the end of the last EA pretreatment were subjected to immunohistochemistry with antibodies against MCPIP1. MCPIP1 expression co-localized with NSE, a neuron-specific marker, implying that MCPIP1 protein expression was increased in neurons after EA treatment (Figure 3c). A co-localization study of MCPIP1 and GFAP, a marker for activated astrocytes, showed a few MCPIP1/GFAP positive cells (Figure 3f). CD11b is a leukocyte-specific receptor and is regarded as a marker for macrophages/microglia. There was no MCPIP1 expression co-localizing in the microglia, as seen by merging of the immunostained images obtained with antibodies against MCPIP1 and macrophage/microglia antigen CD11b (Figure 3i). These observations indicate that MCPIP1 protein is upregulated mainly in neurons and to a lesser extent in astrocytes in the mice brain after EA pretreatment.

### Measurement of MCP-1 and MCPIP1 at later time points after EA pretreatment

**Figure 4:**
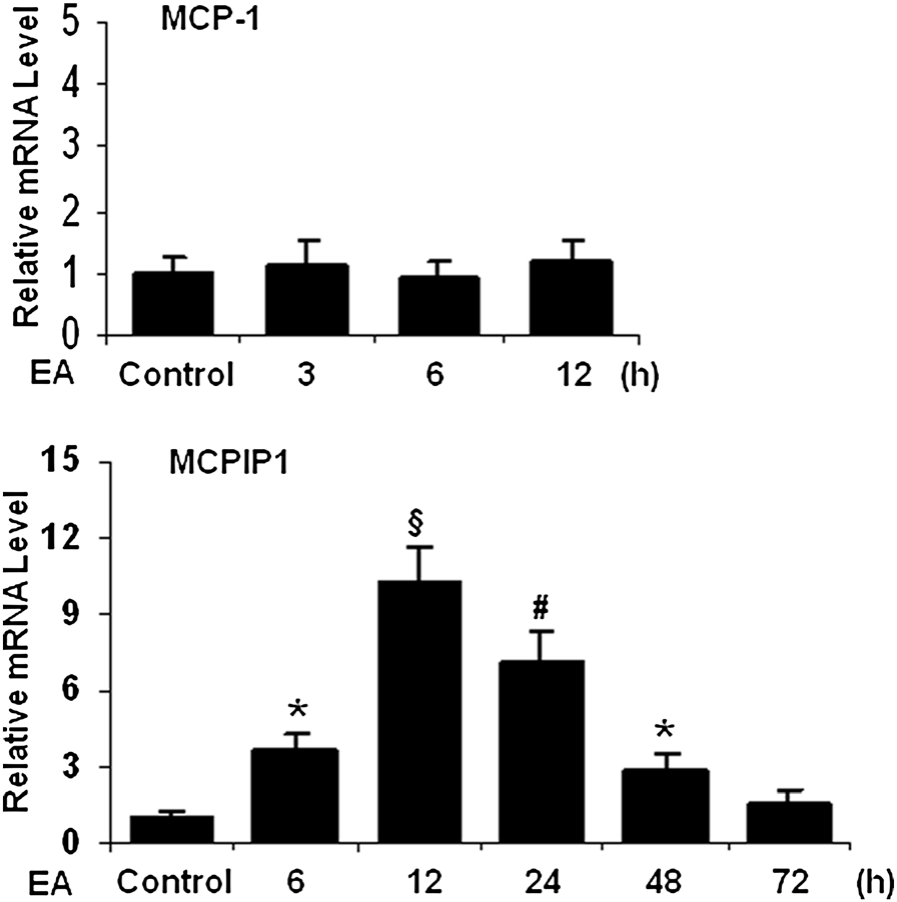
**The expression levels of MCP-1 and MCPIP1 in brain after EA treatment.** The results showed that there were no significant changes in the transcript levels of MCP-1 at 3 h, 6 h and 12 h after EA pretreatment and MCPIP1 mRNA levels decreased at later time points. Values represent mean ± SD, *P <0.05, #*P* <0.01, §*P* <0.001 versus control; n = 6 mice per group. EA, electroacupuncture; MCPIP1, monocyte chemotactic protein-induced protein 1; SD, standard deviation.

To determine the expression of MCP-1 after EA pretreatment we measured MCP-1 mRNA level in mice brain at 3 h, 6 h, and 12 h after EA treatment. The results showed that the expression levels of MCP-1 had no significant changes after EA pretreatment and that MCPIP1 mRNA decreased at later time points (Figure 4).

### Loss of EA pretreatment-induced tolerance to ischemic stroke by MCPIP1 deficiency

**Figure 5:**
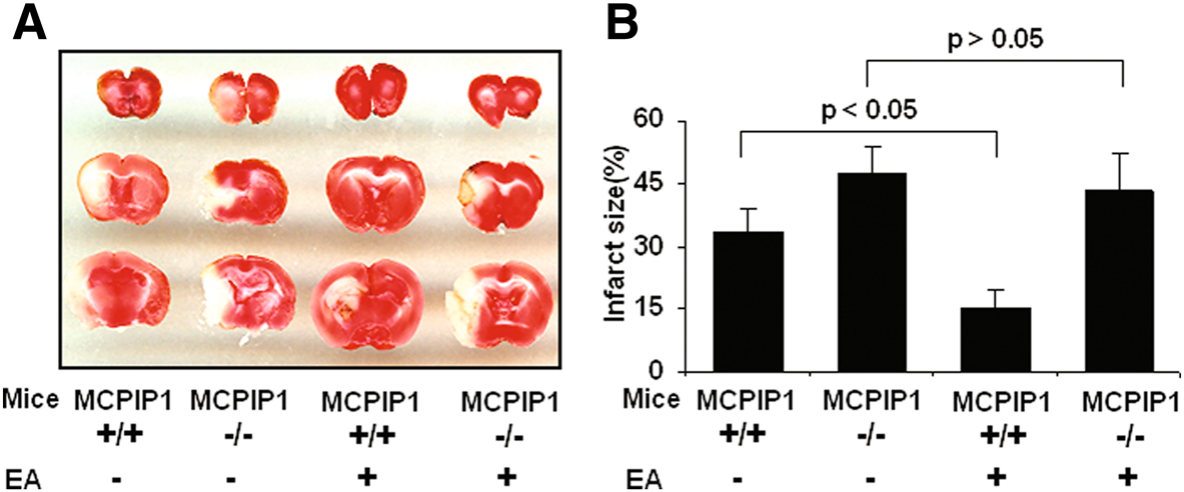
**Reduction in infarct size by EA treatment in wild-type but not in MCPIP1 knockout mice.** The brain infarct size was assessed 48 h after MCAO. **(A)** Infarct images obtained by TTC staining at 48 h after MCAO. The normal tissue was stained deep red and the infarct was stained milky. **(B)** Brain infarcts were quantified as percentage area of ischemic hemisphere. The infarct size of EA-pretreated wild-type mice was significantly reduced compared to that of control. There was no significant difference in brain infarct size between EA-pretreated and control in MCPIP1 knockout mice. Values represent mean ± SD, *P <0.05, n = 10 mice per group. EA, electroacupuncture; MCAO, middle cerebral artery occlusion; MCPIP1, monocyte chemotactic protein-induced protein 1; SD, standard deviation; TTC, 2,3,5-triphenyltetrazolium chloride.

**Figure 6:**
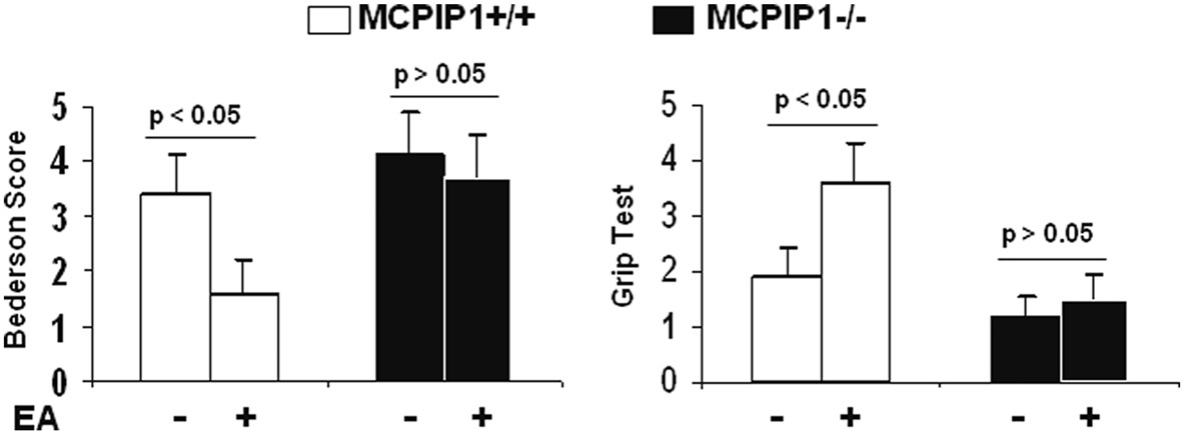
**Protection of neurological function after MCAO by EA treatment in wild-type but not in MCPIP1 knockout mice.** Neurological function assessment was performed 24 h after MCAO. The neurological scores of EA-pretreated wild-type mice were significantly improved compared to that of control. In MCPIP1-deficient mice, there was no significant difference in neurological deficits between the EA-pretreated and the control group. EA, electroacupuncture; MCAO, middle cerebral artery occlusion; MCPIP1, monocyte chemotactic protein-induced protein 1.

We examined the effects of EA pretreatment on ischemic brain infarction. MCPIP1 knockout or wild-type mice were pretreated with EA for two days as described in methods and these mice were subjected to MCAO 24 h after the end of the last EA pretreatment. The brain infarct size was assessed with TTC staining 48 h after MCAO and the results showed that the infarct size of EA-pretreated wild-type mice was significantly reduced compared to that of control. (35.1 ± 5.7% versus 18.2 ± 4.3%, Figure 5A, B). MCPIP1 knockout mice failed to evoke EA pretreatment-induced tolerance compared with that of the control MCPIP1 knockout group without EA treatment (43.2 ± 7.3% versus 47.6 ± 6.9%, Figure 5A, B). There was no significant difference in brain infarct size between EA-pretreated and control in MCPIP1 knockout mice. The neurological functions of mice were determined and the results showed that the neurological scores of EA-pretreated wild-type mice were significantly improved compared to that of control. In MCPIP1-deficient mice, there was no significant difference in neurological deficits between the EA-pretreated and the control group (Figure 6).

### Proinflammatory cytokine expression

**Figure 7:**
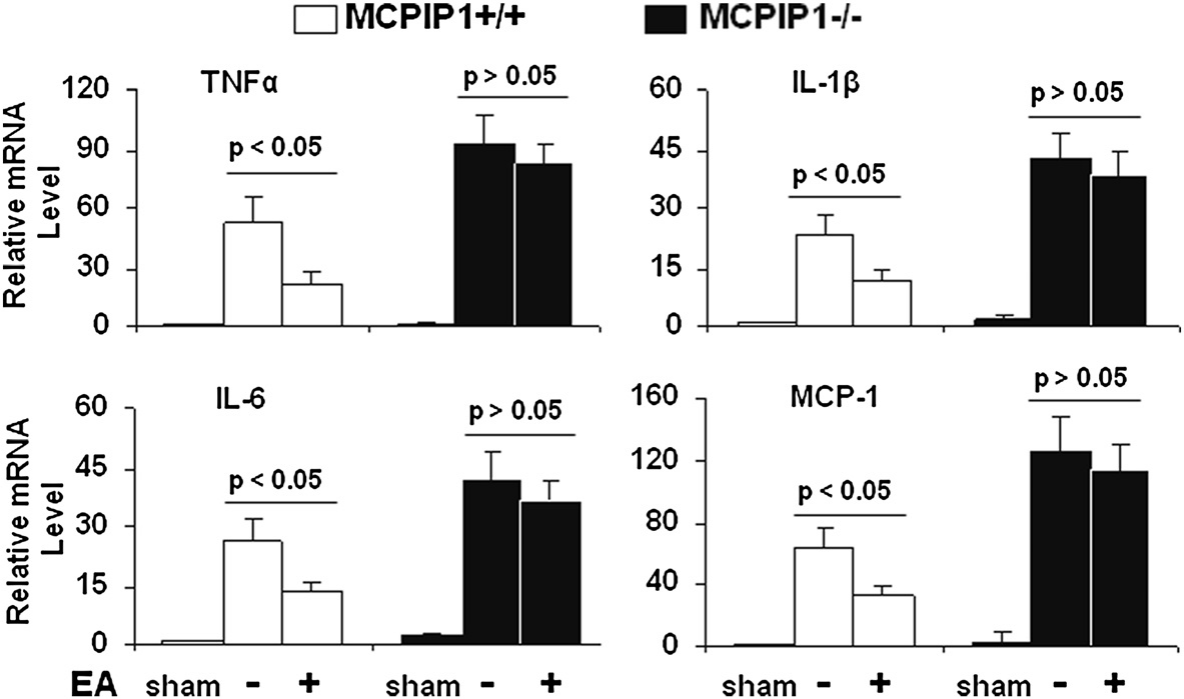
**Reduction in inflammatory cytokine production in ischemic brain by EA treatment in wild-type but not in MCPIP1 knockout mice.** The results showed that the expression levels of TNF-α, IL-1β, IL-6 and MCP-1 were significantly elevated in shams in MCPIP1 knockout mice than that of wild type (for example TNF-α 1 ± 0.27 versus 2.8 ± 0.35) and that the expression levels of TNF-α, IL-1β, IL-6 and MCP-1 were significantly reduced at 24 h after MCAO in EA-pretreated wild-type mice compared to that of control. In the MCPIP1-deficient mice, there was no significant difference in proinflammatory cytokine expression between the EA-pretreated and the control group without EA treatment. Values represent mean ± SD, n = 6 mice per group. EA, electroacupuncture; IL-1β, interleukin-1 beta; MCAO, middle cerebral artery occlusion; MCPIP1, monocyte chemotactic protein-induced protein 1; TNF-α, tumor necrosis factor-alpha; SD, standard deviation.

We examined the expression of proinflammatory cytokine transcript in the ischemic brain of the wild-type and MCPIP1 knockout mice with or without EA pretreatment after MCAO. The results showed that the expression levels of TNF-α, IL-1β, IL-6 and MCP-1 mRNA were significantly elevated in shams in MCPIP1 knockout mice than that of wild type (for example TNF-α: 1 ± 0.27 versus 2.8 ± 0.35) and that the expression levels of TNF-α, IL-1β, IL-6 and MCP-1 were significantly reduced at 24 h after MCAO in EA-pretreated wild-type mice compared to that of non-EA control. In the MCPIP1-deficient mice, there was no significant difference in proinflammatory cytokine expression between the EA-pretreated and the control group without EA treatment (Figure 7).

### Leukocyte infiltration in cerebral brain

**Figure 8:**
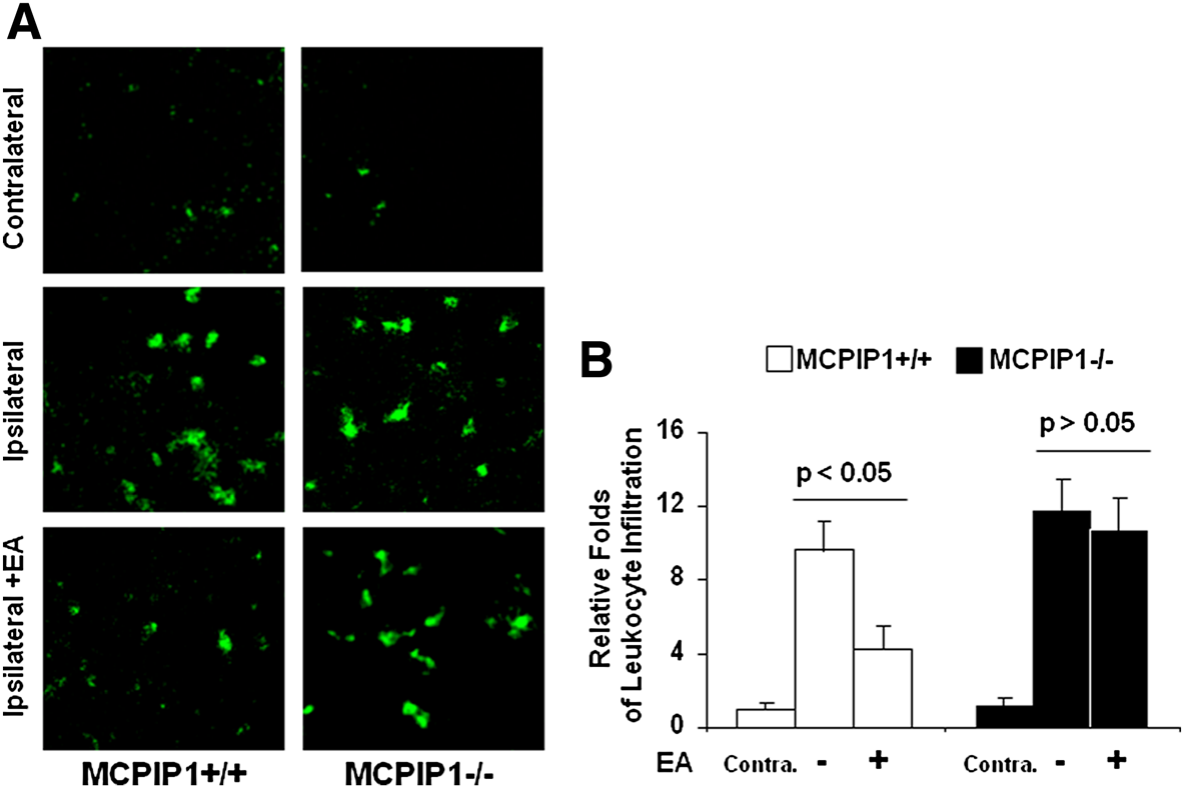
**Leukocyte infiltration in the cerebral cortex.** Reduction of leukocyte infiltration in ischemic cerebral cortex by EA pretreatment in wild-type but not in MCPIP1 knockout mice. **(A)** Immunostaining of leukocyte in the cortex using anti CD45 antibody. **(B)** Qualification of CD45 fluorescence intensity. Each plot is an average of four animals each group. (Magnification 600X.) EA, electroacupuncture; MCPIP1, monocyte chemotactic protein-induced protein 1.

To determine the effect of MCPIP1 on leukocyte infiltration after stroke, we examined leukocyte infiltration in the cerebral cortex in the wild-type and MCPIP1 knockout mice at 48 h after brain ischemia/reperfusion. As illustrated in Figure 8, leukocyte infiltration, visualized with CD45 antibody that specially binds to leukocyte common antigen, was significantly reduced at 48 h after MCAO in EA-pretreated wild-type mice compared to that of non-EA control. In the MCPIP1-deficient mice, there was no significant difference in leukocyte infiltration between the EA-pretreated and the control group without EA treatment (Figure 8).

### Activation of NF-κB signaling pathway

**Figure 9:**
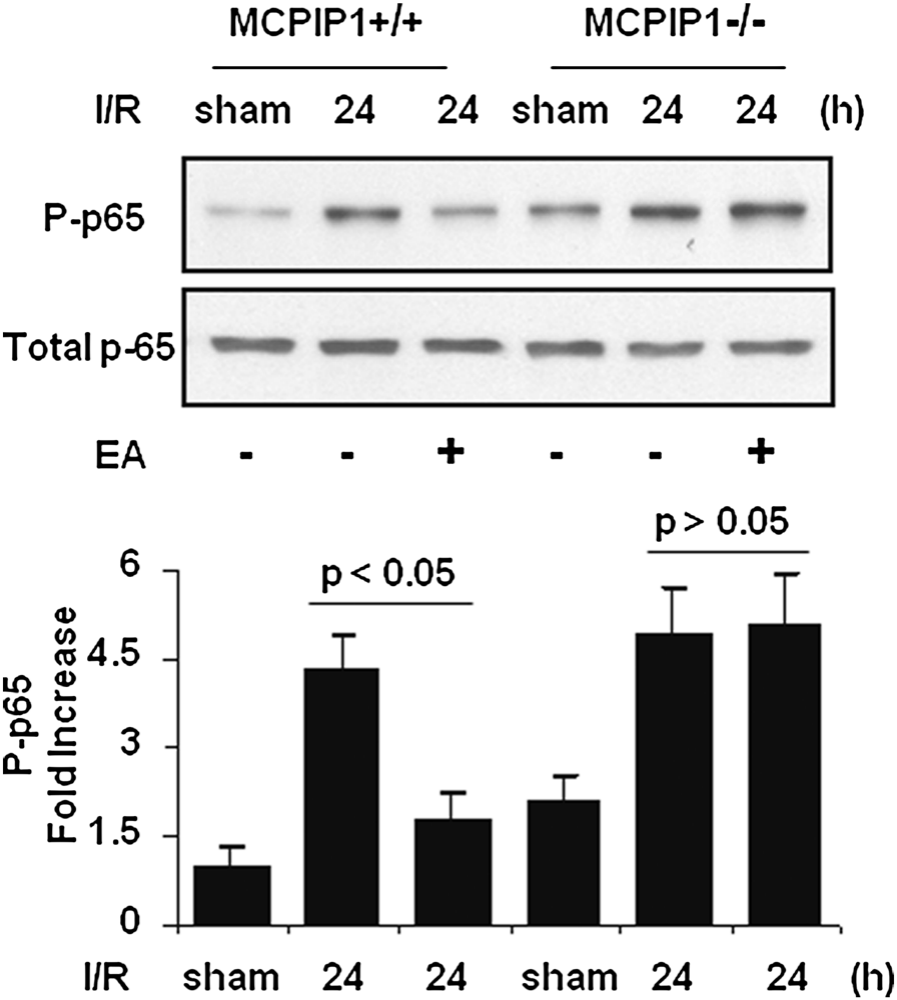
**Inhibition of NF-κB activation in the ischemic brain by EA treatment in the wild-type, but not in MCPIP1 knockout mice.** Activation of NF-κB signaling pathway. A representative western blot shows protein levels of p65 phosphorylation. The phosphorylation of p65 was significantly reduced at 24 h after MCAO in EA-pretreated wild-type mice compared to that of control. In MCPIP1-deficient mice, there was no significant difference in p65 phosphorylation level between the EA-pretreated and the control group without EA treatment. Densitometric analysis was used to quantify phospho-p65 protein levels versus total p65 in three independent western blots and the data are expressed as the normalized folds with respect to sham. Values represent mean ± SD. EA, electroacupuncture; MCAO, middle cerebral artery occlusion; MCPIP1, monocyte chemotactic protein-induced protein 1; NF-κB, nuclear factor-kappa B; SD, standard deviation.

Since activation of the nuclear factor-kappa B (NF-κB) signaling pathway is involved in the production of proinflammatory cytokines, we tested whether EA pretreatment affect NF-κB activation. The results showed that phosphorylation of p65 was significantly reduced at 24 h after MCAO in EA-pretreated wild-type mice compared to that of control. In MCPIP1-deficient mice, there was no significant difference in p65 phosphorylation level between the EA-pretreated and the control group without EA treatment (Figure 9).

## Discussion

Electroacupuncture is a traditional therapy that has been widely applied for treatment of ischemic stroke and many studies have shown that acute EA has therapeutic benefits for stroke patients and in animal stroke models [12,33-38]. Many investigators used five sessions of EA while the others used a single session of EA [29]. In our preliminary experiments, we found that two sessions of EA pretreatment can significantly induce the expression of the MCPIP1 gene and also provide more stable neuroprotective effects on brain infarct volume than that of one session of EA. Therefore, we selected the option of two sessions of EA pretreatment for this study. Although it has been known that EA pretreatment can induce significant tolerance to ischemic brain injury [13,39-42] and inhibit inflammatory responses, such as activation of NF-κB and proinflammatory cytokine generation [21,40], the molecular mechanisms that contribute to brain ischemia tolerance by EA pretreatment are not well understood. The present study is the first to examine the role of MCPIP1 in EA pretreatment-induced cerebral ischemic tolerance. We found that MCPIP1 is significantly induced in mouse brain by EA treatment. This induction of MCPIP1 by the EA treatment was brain-specific as other organs did not manifest such induction. We noticed that there was larger ischemic brain infarct area in MCPIP1-deficient mice compared to that of wild type. In our previous study, we found higher mortality in MCPIP1 knockout mice subjected to focal brain ischemia/reperfusion injury than that of wild type. This finding suggests that MCPIP1-deficient mice are more sensitive to ischemic brain injury than wild type. In the present study, we found that there was significant loss of EA pretreatment-induced ischemic brain tolerance in MCPIP1-deficient mice. These results indicate at least two possibilities: one is that MCPIP1 indeed participates in the EA pretreatment-induced ischemic tolerance; the other is that the preexisting conditions of the MCPIP1 knockout mice may be too detrimental to be overcome by EA pretreatment. Considering the other experimental results in this study, including that MCPIP1 can be significantly induced by EA treatment in the brain, and that MCPIP1 has been identified as an important inducible anti-inflammatory regulator in stroke pathophysiology [28], it would appear more likely to that MCPIP1 actually participates in the EA pretreatment-induced ischemic tolerance. The role of MCPIP1 as a mediator of EA-induced tolerance of ischemic brain damage is consistent with the well-known role of inflammatory processes in the brain damage in ischemic stroke [4] and the anti-inflammatory properties of MCPIP1 [24,25]. Thus, we conclude that MCPIP1 is involved in EA pretreatment-induced delayed ischemic stroke tolerance by its anti-inflammatory activities.

It is well known that proinflammatory gene expression and leukocyte infiltration contribute to stroke damage [5,43,44]. During ischemia, cytokines, such as TNF-α, IL-1β, IL-6, and chemokines such as cytokine-induced neutrophil chemoattractant (CINC) and MCP-1 are produced by a variety of activated cell types, including endothelial cells, microglia, astrocytes and neurons [46]. EA pretreatment can induce significant tolerance to ischemic brain injury and inhibit inflammatory responses such as activation of NF-κB and proinflammatory cytokine generation. However, the molecular mechanisms that mediate the beneficial effects in EA pretreatment-induced ischemic tolerance remains poorly understood. In this study, we observed that TNF-α, IL-1β, IL-6 and MCP-1 expressions were significantly reduced at 24 h after MCAO in EA-pretreated wild-type mice compared to that of control. In MCPIP1-deficient mice, there was no significant difference in proinflammatory cytokine expression between the EA pretreatment and the control group without EA treatment. It has been demonstrated that leukocyte infiltration into the ischemic brain in transient MCAO models is prominent and that anti-leukocyte strategies (including anti-adhesion molecule strategies) have generally proven to be significantly effective in animal stroke models of transient ischemia. In the present study, we observed that the infiltration of leukocytes was significantly reduced at 48 h after MCAO in EA-pretreated wild-type mice compared to that of non-EA control mice. In the MCPIP1-deficient mice, there was no significant difference in leukocyte infiltration between the EA-pretreated and the control group without EA treatment.

It has been established that EA pretreatment can induce ischemic tolerance. Less well studied is what mediates such beneficial tolerance in the preconditioning process. If this mediator is absent, EA pretreatment-induced tolerance would not occur. We conclude that MCPIP1 is such a mediator that is involved in EA pretreatment-induced tolerance. When MCPIP1 is deficient, EA-induced tolerance is reduced and results in higher level of proinflammatory cytokines and greater infiltration of leukocytes in ischemic brain.

How MCPIP1 regulates these inflammatory cytokine responses remains to be fully elucidated. It has been reported that MCPIP1 might be functioning as an RNase to promote the degradation of mRNA for inflammatory cytokines, such as IL-6 and IL-1β [25]. We have found that MCPIP1 can also act as a deubiquitinase to negatively regulate NF-κB signaling by targeting TNF receptor-associated factors (TRAFs) [24,45], which suggests that MCPIP1 may control inflammatory response by multiple mechanisms. Activation of NF-κB signaling pathways leads to inflammatory cytokine production. In this study, we found that that phosphorylation of p65 was significantly reduced at 24 h after MCAO in EA-pretreated wild-type mice compared to that of control. In MCPIP1-deficient mice, there was no significant difference in p65 phosphorylation level between the EA-pretreated and the control group without EA treatment. Our study suggests that increased activation of NF-κB signaling pathways in MCPIP1 knockout mice leads to increased proinflammatory cytokine production.

The main purpose of this study was to determine the participation of the MCPIP1 gene expression in EA pretreatment-induced ischemic stroke tolerance. Whether EA treatment after stroke provides therapeutic benefits, and if MCPIP1 might be involved in such possible benefits, require exploration. Since EA-induced MCPIP1 expression is mostly in the neurons, mice with neuron-specific MCPIP1 deficiency might provide a suitable model to explore the role of MCPIP1 in stroke-induced damage. Efforts are under way to explore such possibilities.

## Conclusions

Based on the data, we conclude that MCPIP1 induction mediates the EA-induced delayed tolerance of ischemic brain injury and that MCPIP1 is involved in EA pretreatment-induced brain ischemia tolerance.

## Competing interests

The authors declare they have no competing interests.

## Authors’ contributions

ZJ and JL designed the experiments, performed all experiments, analyzed the data, generated the figures, and wrote the manuscript. JW did parts of the animal surgery and performed the experiments. PEK provided advice in the design of the study and in interpreting of data and critically read and corrected the manuscript. All authors have read and approved the final manuscript.
